# Tuning the 3D Printability and Thermomechanical Properties of Radiation Shields

**DOI:** 10.3390/polym13193284

**Published:** 2021-09-26

**Authors:** Zachary Brounstein, Jianchao Zhao, Jeffrey Wheat, Andrea Labouriau

**Affiliations:** 1 Los Alamos National Laboratory, Los Alamos, NM 87545, USA; zrbrounstein@lanl.gov (Z.B.); jczhao@umich.edu (J.Z.); jwheat058@gmail.com (J.W.); 2 Department of Nanoscience and Microsystems Engineering, University of New Mexico, Albuquerque, NM 87131, USA; 3 Department of Chemical Engineering, University of Michigan, Ann Arbor, MI 48109, USA

**Keywords:** direct ink writing, radiation shielding, thermomechanical properties, 3D printability, neutron radiography, siloxanes, inorganic fillers, ionizing radiation

## Abstract

Additive manufacturing, with its rapid advances in materials science, allows for researchers and companies to have the ability to create novel formulations and final parts that would have been difficult or near impossible to fabricate with traditional manufacturing methods. One such 3D printing technology, direct ink writing, is especially advantageous in fields requiring customizable parts with high amounts of functional fillers. Nuclear technology is a prime example of a field that necessitates new material design with regard to unique parts that also provide radiation shielding. Indeed, much effort has been focused on developing new rigid radiation shielding components, but DIW remains a less explored technology with a lot of potential for nuclear applications. In this study, DIW formulations that can behave as radiation shields were developed and were printed with varying amounts of porosity to tune the thermomechanical performance.

## 1. Introduction

As the world advances in additive manufacturing and materials science, many forms of 3D printing are being researched with regard to materials development and macroscopic part fabrication. Indeed, major progress in vat polymerization [1,2], selective laser melting [3,4], and direct ink writing (DIW) [5,6,7] has demonstrated the variety of means in which advanced composites can be used to construct geometries and structures that traditional manufacturing techniques have difficulty fabricating. DIW 3D printing, a technology under the ISO/ASTM 52900:2015 category of material extrusion, includes ink jet printing [8,9,10], micropen writing [11,12], fused filament fabrication (FFF) [13,14,15], hot-melt extrusion [16,17], and robocasting [18,19] and is especially useful because of the wide array of material selection available and the continued development which increases the capabilities of these techniques. DIW, in particular, is especially suitable for advanced materials capabilities due to its range of ink formulations and versatility in part extrusion and curing. Customarily, DIW refers to the 3D printing technique where a shear-thinning fluid, ink, or paste is extruded through a nozzle and possesses a high enough storage modulus to build a part layer by layer. Additionally, these extrusion methods also allow for numerous hardening regimes such as UV curing, elevated temperature curing, freeze drying, and other means, which increases the possible range of new materials that can be 3D printed. Examples include aerogels and foams developed from ceramics and carbonaceous materials [20,21,22,23], synthetic bone and osteoinduction scaffolds [24,25,26,27], smart magnetoresponsive devices [28,29,30], and more. Thus, DIW lends itself to immense materials development exploration and can result in a variety of specialty parts for many applications.

One area that would benefit from the advances in manufacturing science that DIW offers is that of radiation shielding. Since commercial nuclear power has become more widespread, ionizing radiation in the form of gamma rays and neutrons has been attenuated with large blocks of concrete, lead, or boron [31,32,33]. Additionally, some high-Z elements such as gadolinium and tungsten were and are still used for gamma radiation shielding [34,35]. Recently, more precise radiation shielding materials have been developed such as glasses and amorphous alloys for use in other nuclear technologies such as radiation protection and medicine [36,37,38]. The advancement in this area of materials development has also occurred with 3D printing technology, where filaments for FFF and inks for DIW have been created in contemporary research [14,39,40,41,42,43,44]. Indeed, the merging of the two fields of nuclear technology and advanced manufacturing proves especially prolific and rewarding due to the unique part fabrication that 3D printing offers, where commercial entities have begun selling radiation shielding material specifically for additive manufacturing technologies. Although at the nascent stage where much materials research and development needs to occur, this nevertheless represents a growing endeavor due to the continued and increasing interest in nuclear energy, nuclear medicine, nuclear waste storage, high-energy physics, and space exploration [35,45,46,47,48].

With regard to 3D printing, there are two large factors that imbue a final product with its material properties. These are the basic characteristics of the constituent components and the auxiliary characteristics of the macroscopic structure, which provide the essential and specialty qualities of a material, respectively. The former factor is shared with traditional manufacturing techniques, but the latter is given by how the material is printed with whichever 3D printing technology is used. Thus, besides the rapid prototyping and part development that are manufacturing advantages, 3D printing technologies offer hierarchical structural properties. Examples of these can be geometries that are impossible or near impossible to fabricate with traditional manufacturing techniques, where shape, hollowness, and porosity combine to allow parts with precise customized material properties [49,50,51,52,53,54]. By controlling the shape, hollowness, and porosity of an additively manufactured product, transport properties such as diffusion, thermal conductivity, and mechanical response can be governed [55,56].

In this work, various ink formulations were developed for DIW 3D printing that behave as radiation shields and possess tunable thermomechanical properties. Using a base formulation with two siloxane copolymers and a platinum catalyst that enables a curing reaction based on elevated temperatures, fillers such as fumed silica, tungsten, tungsten (VI) oxide, gadolinium (III) oxide, and boron were incorporated into 3D printable inks. Rheological properties of a representative sample of the inks were evaluated, and an empirical relationship was developed that provides a model for the upper limit on the spacing ratio, a lattice parameter, during 3D printing. Compressive strain and thermal conductivity measurements of the printed structure demonstrated that there is a correlation between porosity and thermomechanical properties. Additionally, thermal stability experiments showed that the radiation shielding ink formulations can be used in environments at much higher temperatures than a regular ink which controls for just rheology. Neutron radiography experiments provided evidence that the printed formulations attenuate ionizing radiation. Finally, heterogeneous printed parts were produced using two different ink formulations to demonstrate that the capabilities offered by this technology allow for greater materials development precision than traditional manufacturing.

## 2. Experimental

### 2.1. Materials

The siloxanes that composed the polymer network included vinyl-terminated (4–6% diphenylsiloxane)-dimethylsiloxane copolymer (PDV−541) and trimethylsiloxy-terminated methylhydrosiloxane-dimethylsiloxane copolymer (HMS 301), both from Gelest (Gelest, Inc., Morrisville, PA, USA). A cure inhibitor in the form of 1-ethynyl-1-cyclohexanol was used (Sigma Aldrich, 99%) (Millipore Sigma, St. Louis, MO, USA), and crosslinking was induced with a high-temperature platinum catalyst (platinum carbonyl cyclovinylmethylsiloxane complex; 1.85–2.1% Pt in cyclomethyl vinyl siloxanes) (Gelest, SIP6829.2). An OH-functionalized fumed silica (Evonik Aerosil 300) (Evonik Industries AG, Essen, Germany) and PDMS-functionalized fumed silica (CAB-O-SIL TS-720) (Cabot Corporation, Boston, MA, USA) were incorporated into the polymer matrix. Boron, tungsten, tungsten (VI) oxide, and gadolinium (III) oxide powders, supplied by American Elements (American Elements, Los Angeles, CA, USA), were used as fillers in the formulation. Isotopically enriched B10 was supplied by 3M (3M Company, Saint Paul, MN, USA). Isopropanol (IPA) was supplied by Thermo Fisher Scientific (Thermo Fisher Scientific, Waltham, MA, USA). Ultra-high purity nitrogen was supplied by Airgas (Airgas, Padnor, PA, USA).

### 2.2. Formulation Development and 3D Printing

Stable and 3D-printable inks depend on the formulation exhibiting specific rheological properties. In particular, the ink needs to exhibit shear thinning; it must flow when a force is applied and remain stiff otherwise. This is especially the case once the ink is printed into a part where it must support its own weight and not collapse. This specific non-Newtonian rheological characteristic was imparted to the polymer matrix by incorporating fumed silicas, whereby varying the amount of fumed silicas allowed the rheological properties to be tuned. PDMS-functionalized fumed silica, referred to in this study as TS720, acted as an inert filler which solely provided shear thinning characteristics. OH-functionalized silica, referred to in this study as A300, behaved as a filler that could form hydrogen bonds, thus providing both shear thinning characteristics and increasing the amount of physical crosslinks in the network. The vinyl-terminated copolymer, referred to in this study as PDV, was always added in a 9:1 w/w ratio with the trimethylsiloxy copolymer, referred to in this study as HMS, which was found in previous work to attenuate radiolysis and prevent crystallization [39,57]. Metal and ceramic fillers were sieved (Gilson Company, Inc., Lewis Center, OH, USA) so the particle size distributions had an upper limit of 53 µm.

To begin producing formulations, an ink containing only silica as a filler was first developed and studied. Increasing the weight percent of silica resulted in a more viscous ink. Due to the nature of DIW 3D printing, a requirement of the formulation is that it remains stiff and rigid while under the force of gravity, but when a sufficiently high force is applied, it becomes liquid-like and flows. It was found that when the OH-functionalized fumed silica (A300) content was under 10 wt%, the DIW formulation flowed even without an applied force, thus making it unusable for printing. At 10 wt%, the ink was viscous enough and could be printed successfully. Rheological experiments were performed on this recipe to determine its equilibrium storage modulus, yield stress, and flow point. After this formulation was characterized, other fillers were incorporated to develop new recipes. Using amounts of 50 wt% non-SiO_2_ filler, the fumed silica content was modified to obtain similar rheological properties to the 10 wt% silica recipe.

Although the PDMS-functionalized silica (TS720) increased the rheological properties of the ink enough to where printing was possible, the printed layers of the final part had an excessive amount of slumping and thickness deviations. Additionally, larger amounts of TS720 were required to achieve adequate rheology for printing, which led to fewer amounts of other fillers added and to the inks being too dry to adhere onto the glass plates where printing occurred. This was remedied with IPA, where, by adding 10–20 wt%, the solvent swelled the polymer matrix and allowed for high amounts of filler to be incorporated and produce an adequate print. Unfortunately, during the high-temperature curing process at 150 °C, cracks were formed in the final part, and it appeared that while varying the amounts of all the components led to more or less defects, flaws were always present in the end. This is inferred to be due to the IPA evaporating and leaving mesoscale pores of non-uniform size and morphology within the struts, which was confirmed when viewed under a confocal digital microscope. While this hierarchical porous architecture is an area of further research, and continued pursuit could be beneficial towards other applications, this study wanted to focus on denser printed pads for radiation shielding. As such, TS720, with its lack of hydrogen bonding, was not used further in this study. Moving forward, A300 was the silica of choice, which did not require the use of IPA.

Once the resins were formulated and mixed for DIW printing, they were transferred into a metal syringe (EMO-XT printer head, Hyrel 3D) (Hyrel 3D, Atlanta, GA, USA) and then centrifuged at 2000 rpm for 1–2 min to remove any air bubbles. A MATLAB script was created to generate a Gcode with varying amounts of spacing and geometries. Repetrel software (Hyrel 3D) was used to control the printer and ran at a travel rate at 2250 mm/min, with the material flow rate at 150 pulses/μL onto a glass substrate from the build stage. The geometries of the 3D parts were disks possessing a diameter of 5 cm and consisted of eight layers with each layer organized in a faced-centered tetragonal (FCT) structure. Four different spacings between the printed struts (500 μm, 750 μm, 1000 μm, and 1500 μm) were used for tuning the thermomechanical properties and were cured in an oven at 150 °C for 2 h.

### 2.3. Material Characterization Techniques

Rheological experiments on a representative sample of ink formulations were conducted on a TA Discovery Series Hybrid Rheometer DHR-3 (TA Instruments, New Castle, DE, USA) using a 25 mm cross-hatched parallel plate fixture geometry. Strain sweeps were performed from 0.001% to 10% strain at an angular frequency of 10 rad/s to determine the extent of the linear viscoelastic region of the samples. The sample containing boron and gadolinium (III) oxide (B/Gd_2_O_3_) was run from 0.00025% to 0.5% at an angular frequency of 1 rad/s. Stress sweeps were performed from 10 to 10,000 Pa at an angular frequency of 10 rad/s. The equilibrium storage moduli ${G^{\prime }}_{eq}$ for the samples were determined from the plateau of the stress sweeps in the linear viscoelastic region. The yield stress $\sigma_{y}$ was determined from the intersection of lines formed from the storage moduli of the linear viscoelastic region and the beginning of the nonlinear viscoelastic region. The flow point is the stress at which the storage and loss moduli cross or intersect. Based on calibration testing from the manufacturer and comparative measurements, the error associated with this instrument and the resulting values are less than 1%.

Uniaxial compression tests were performed using an INSTRON^®^ 3343 Low-Force Testing System (Instron, Norwood, MA, USA) with the BlueHill Universal software. Each printed sample was compressed for 4 cycles at a rate of 0.05 mm/sec through the stress range from 0 to 0.4 MPa. The cyclic stress–strain curve and the Young’s modulus were reported from the fourth cycle to minimize the Mullins effects. Based on calibration testing from the manufacturer and comparative measurements, the error associated with this instrument and the resulting values are less than 1%.

Thermal conductivity was performed by a TA Fox 50 Heat Flow Meter (TA Instruments, New Castle, DE, USA). Compressed air flowed to the instrument at 60 psi in order to pneumatically compress the samples between two thermally responsive plates. The protocol included nine temperature regimes where the upper and lower plates had a temperature difference of 10 °C, starting with the plates equilibrating to 20 °C and 10 °C, and ending with the plates equilibrating to 100 °C and 90 °C. Based on calibration testing from the manufacturer and comparative measurements, the error associated with this instrument and the resulting values are less than 3%.

Thermogravimetric analysis (TGA) was performed on a TA Q Discovery 2000 series TGA instrument (TA Instruments, New Castle, DE, USA). The protocol included ramping the surrounding temperature of a sample weighing approximately 5 mg from 25 to 750 °C at a heating rate of 10 °C/min. Ultra-high purity nitrogen flowed across the sample at a rate of 40 mL/min. The onset of thermal degradation *T_d5%_* was taken as the temperature at which a sample lost 5% of its mass (or possessed 95% of its mass remaining). The decomposition temperatures *T_dMax_* are those temperatures at which the derivative TGA curves (DTGA) are at a local maximum. The final mass *m_f_* is the residual mass of the sample after the temperature protocol has been executed. Based on calibration testing from the manufacturer and comparative measurements, the error associated with this instrument and the resulting values are less than 1%.

The magnified and cross-section views of the sample images were taken from a confocal digital microscope (Keyence VHX-6000) (Keyence Corporation, Osaka, Japan) and micro X-ray fluorescence (MXRF) (Bruker M4 Tornado) (Bruker Corporation, Billerica, MA, USA). Magnifications of 20×, 30×, and 100× were used to investigate the network of the resulting 3D-printed pads. Measurements of the printed struts were obtained from the Keyence analysis software. Elemental color maps were generated from MXRF images using the instrument software. The acquisition parameters included an X-ray tube operating at 50 kV and 200 μA, a spectrometer operating at 40 keV and 130 kcps, a spot size of 20 μm, a dwell time of 5 ms per pixel, and a step size of 10 (cross-section) by 20 μm (top down).

Advanced neutron radiography was performed at the Los Alamos Neutron Science Center (LANSCE) via energy-resolved neutron imaging (ERNI). Neutrons in the energy range from 0.001 to 100 eV (epi-thermal to thermal) pulsed at 20 Hz at printed samples, which were in front of an ultra-fast MCP-Timpix neutron imaging detector. Details can be found in our previous work [39]. In the resulting radiographs, lighter images correspond to more neutrons hitting the detector, whereas darker images correspond to less neutrons hitting the detector, providing a qualitative measure of the neutron attenuation abilities of the printed radiation shields. It should be noted that the neutron background at thermal energies was not well characterized during the ERNI experiments. Without proper background characterization, quantitative comparisons are more difficult to perform. That stated, the qualitative assessment using this technique provides ample evidence of successful neutron attenuation.

## 3. Results and Discussion

### 3.1. Rheology

When developing ink formulations with A300, incorporating a single metal or ceramic filler that was denser than boron (tungsten, tungsten (VI) oxide, and gadolinium (III) oxide) at 50 wt% was printable with 4.5 wt% fumed silica. To confirm that these formulations matched the 10 wt% silica formulation, rheological experiments were performed on the tungsten and tungsten (VI) oxide recipes. These strain and stress sweep experiments validated that the amount of silica and other fillers demonstrated similar rheological properties to the 10 wt% silica formulation. As boron is less dense than the other metals and ceramics, a recipe containing 50 wt% boron will correspond to a greater volume percent than the others. Thus, the silica content needed to be further reduced. It was found that incorporating 1.5 wt% A300 with boron led to the desired rheological properties. Following the success of developing and 3D printing the boron formulations, combinations of the metals and ceramics were made into recipes. Specifically, B/Gd_2_O_3_ and B/Gd_2_O_3_/WO_3_ formulations were created with a combined 70 wt% non-SiO_2_ filler content. A similar silica content was found to result in successful recipes, and rheological experiments were performed on the 40/30 wt% B/Gd_2_O_3_ formulation. The equilibrium storage modulus was found to be greater than the others; however, the linear viscoelastic region, yield stress, and flow point were in similar ranges, and the ink could be successfully 3D printed. The formulations developed for this study, along with their filler weight and volume percent, and density, are presented in Table 1. The densities of the inks $\rho_{ink}$ were determined from the weight fractions of the constituent components $w_{i}$ and their individual densities $\rho_{i}$ using Equation (1).

(1) $$\rho_{ink}=\frac{1}{\sum_{i}\frac{w_{i}}{\rho_{i}}}$$

**Figure 1:**
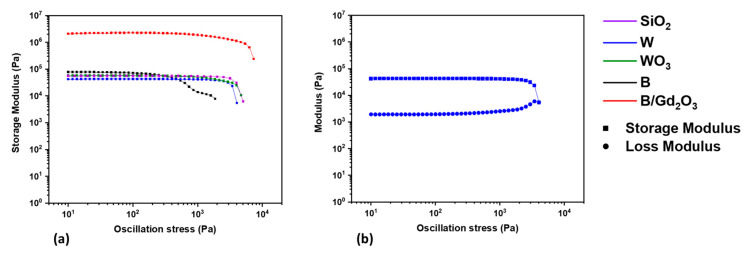
Rheological experiments showing (**a**) the storage modulus of the linear viscoelastic region and beginning of the nonlinear viscoelastic region of a representative sample of ink formulations, and (**b**) the loss and storage moduli of the W formulation until the flow point.

Strain and stress sweep rheological experiments were performed to determine when the ink formulations cross from the linear viscoelastic region to the nonlinear viscoelastic region. The storage moduli, taken during the stress sweep experiments, of the inks are shown in Figure 1a,b, which present the storage and loss moduli of the W formulation taken from the stress sweep experiment, where the flow point, which is the stress at which the storage and loss moduli cross or intersect, can be observed. The equilibrium storage modulus, yield stress, and flow point values for the ink formulations tested in rheology experiments are presented in Table 2. The values demonstrate similar rheological properties and an ability of the inks to be successfully 3D printed with the motor capabilities of the DIW printer used.

### 3.2. 3D Printing

**Figure 2:**
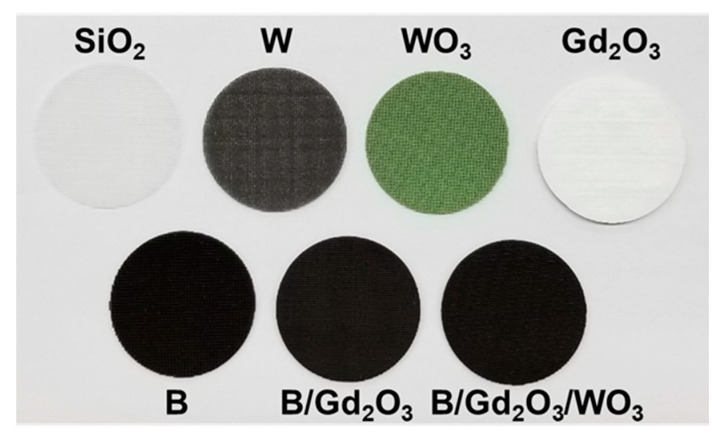
3D-printed cylinders of the ink formulations detailed in this study.

Using the ink formulations, cylinders in the form of disks were 3D printed with increasing amounts of introduced porosity. Figure 2 shows 3D-printed cylinders of the formulations detailed in this study. Printing with well-defined porosity was accomplished by varying the spacing ratio η=Ld, which is the ratio of the center-to-center distance between adjacent struts *L* to the diameter of the printed struts *d*. Cylinders were printed with spacing ratios of 2, 3, 4, and 6. As the printing nozzle measured 250 µm in diameter, which corresponds to the diameter of the struts, the spacing ratio corresponds to center-to-center distances between adjacent struts of 500, 750, 1000, and 1500 µm.

**Figure 3:**
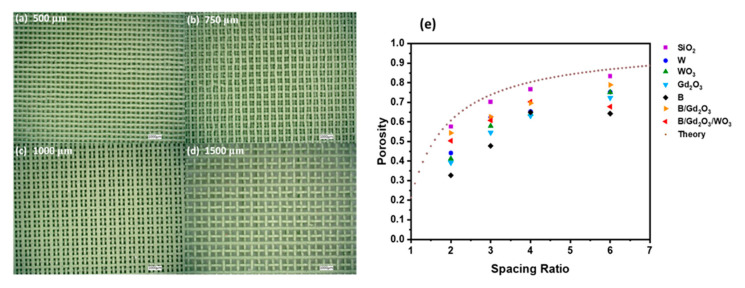
Porosities of the printed formulations at varying spacing ratios, with microscopy images of WO_3_ at (**a**) 500 µm or *η* = 2; (**b**) 750 µm or *η* = 3; (**c**) 1000 µm or *η* = 4; and (**d**) 1500 µm or *η* = 6; and (**e**) comparing the porosities of the printed samples along with the theoretical 3D-printed porosities.

Considering the theoretical model relating the porosity of a structure with an FCT geometry to the spacing ratio (presented in Supplemental Information), taking the limit yields a theoretical porosity as a function of the spacing ratio, which is presented as Equation (2). Using the calculated densities of the ink and measuring the densities of the 3D-printed cylinders structured with FCT geometries $\rho_{structure}$, the actual porosities of the cylinders were calculated using Equation (3). A graph of the porosity of the printed parts plotted against their spacing ratio is presented in Figure 3 along with the theoretical porosity.

(2) $$\varphi_{FCT}^{\infty }=\underset{n\to \infty }{\lim}\varphi_{FCT}=1-\frac{\pi d}{4L}=1-\frac{\pi }{4\eta }$$

(3) $$\varphi_{FCT}=\varphi_{structure}=1-\frac{\rho_{structure}}{\rho_{ink}}$$

### 3.3. Rheology–Printability Relationship

**Figure 4:**
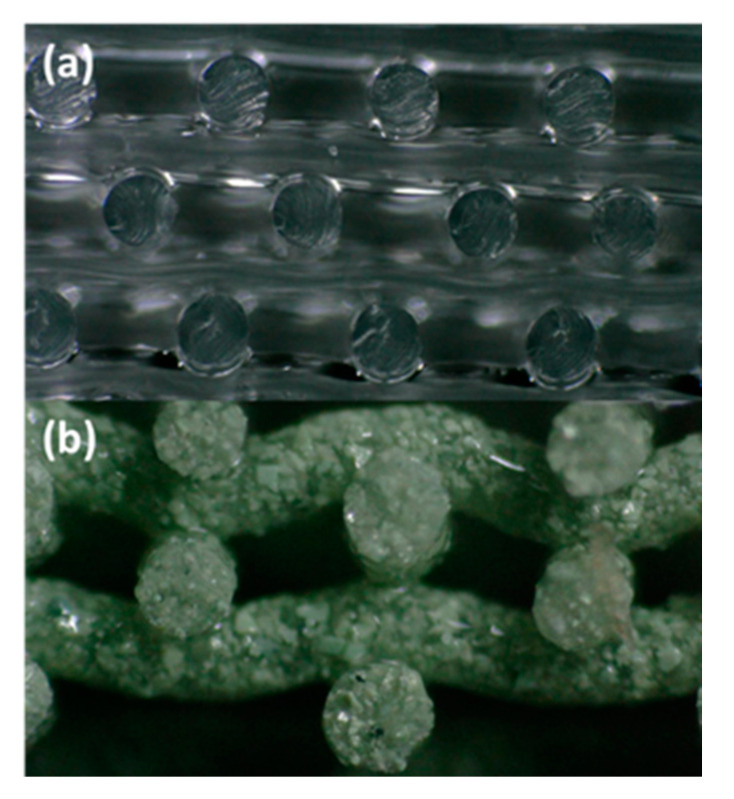
Side views comparing deviations in printed (**a**) SiO_2_ and (**b**) WO_3_ samples.

It is worth pointing out that all the cylinders exhibit porosities less than the theoretical porosity, thus demonstrating that Equation (2) is an upper limit. This can be observed more distinctly when comparing the side views of some of the printed cylinders. An example is Figure 4, which shows side views of printed SiO_2_ and WO_3_ samples. Notice in Figure 4a that the SiO_2_ lattice structure is aligned such that there do not appear to be deviations, while in Figure 4b, the WO_3_ lattice has some bending or deflections in the printed struts.

Previous researchers have attempted to capture this phenomenon with a variety of theoretical, phenomenological, and empirical models, where the properties of the ink formulation relate to the printed lattice structure. Smay et al. correlated deflection in a strut with the distance between struts across a variety of ink formulations at different pH values and provided a criterion for the storage modulus [50]. The relevant values for the ink formulations in this study were placed in this criterion, which was not found to accurately predict the printing behavior. M’Barki et al. also developed a model that incorporated other variables such as the yield stress, print height, and capillary forces; however, it did not relate these to the spacing ratio of the lattice structure [58]. Chan et al. tested the rheology of some inks against their printability and came to the same conclusion [49] for the two models studied. To move the discussion forward, Chan et al. proposed an empirical relation between the equilibrium storage modulus, recovery of the storage modulus after shearing, and the yield stress. A constant derived from a linear discriminant function was able to separate the ink formulations into those that slumped upon printing and those that printed well; however, this model does not correlate the ink rheology with the printability of a lattice structure based on the spacing ratio. Therefore, the ink formulations in this study were used to inform another model that can relate rheology and printability. 

To understand the deflection behavior observed in Figure 4b, an appropriate model can be constructed by considering the simplified scenario of a beam with supports on either end. This is a standard problem in civil engineering, where it can be assumed that a beam is supported by the two orthogonally printed struts below it, and there are no layers printed above. Thus, the length of the strut is the center-to-center distance between the struts below L=ηd, and the only acting force F is gravitational. The deflection δ that the beam experiences is a function of the force, beam length, Young’s modulus of the ink E, and the moment of inertia I of a cylinder rotated about its axial direction, which is shown in Equation (4). Considering the deflection as a percentage of the beam diameter δ=αd and rearranging all the numerical constants on one side and all the variables on the other side result in Equation (5).

(4) $$\delta =\frac{FL^{3}}{48EI}=\frac{\rho g\frac{\pi }{4}d^{2}L^{4}}{48E\frac{\pi }{64}d^{4}}$$

(5) $$3\alpha =\frac{\rho g\eta^{4}d}{E}=\left(E^{-1}\rho g\right)\left(\eta^{4}d\right)$$

This model demonstrates which material properties are important towards understanding beam deflection with supports on either side. Building up this model to describe an entire printed lattice can be conducted in a phenomenological way by utilizing similar variables to those in Equation (5); however, instead of evaluating the deflection of a single beam, the relationship between the ink properties and overall lattice structure can be investigated. As Young’s modulus is a measure of an elastic material and the ink is viscoelastic, conceptually equivalent material properties that describe the system should be used instead. Young’s modulus is the stiffness of a material in the linear viscoelastic region, meaning a commensurate property to determine the stiffness of the inks would be the equilibrium storage modulus. Indeed, others have shown that the storage modulus and yield stress are correlated with the layer shape retention of deposited layers [44,51]. Conceptually, this makes sense, where the lattice structure and its deviations should be able to be represented by the ink’s equilibrium storage modulus and yield stress, which describe how an ink prints and whether it can support itself. Using these, a variable referred to as the ink parameter was defined, ${Κ}_{ink}={G^{\prime }}_{eq}\sigma_{y}{}^{-2}\rho_{ink}g$. To incorporate the printed structure itself, a variable called the lattice parameter was defined, $\Psi_{lattice}=\eta^{4}d$. The product of these two variables is a dimensionless quantity called the structure parameter in this study, which is given as Equation (6). This product, being dimensionless and of similar form to Equation (5), can provide an empirical determination on how the spacing ratio in a lattice and general printing properties can be related to the ink formulation attributes.

(6) $${Κ}_{ink}\Psi_{lattice}=\left({G^{\prime }}_{eq}\sigma_{y}{}^{-2}\rho_{ink}g\right)\left(\eta^{4}d\right)$$

**Figure 5:**
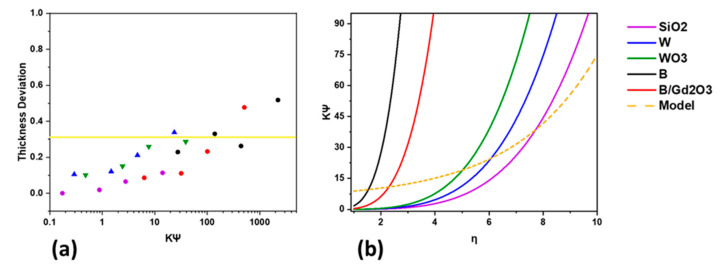
Determining how the structure parameters are related to spacing ratios, where (**a**) the thickness deviation is plotted against ${Κ}_{ink}\Psi_{lattice}$, with a yellow line at the one standard deviation mark denoting whether a print was acceptable, and (**b**) ${Κ}_{ink}\Psi_{lattice}$ plotted against the spacing ratio along with the model presenting the upper bound for the structure parameters.

Notice from Figure 3 that the SiO_2_-formulated cylinders are closest to the theoretical limit. Thus, the height of the printed SiO_2_ cylinders was used as an ideal for comparison purposes. Defining the thickness deviation of a printed cylinder t′=1−hink,ηhSiO2,η in terms of the SiO_2_ print with the same spacing ratio, all the inks that had their rheological properties tested can be compared. Figure 5a presents a graph plotting the thickness deviations against the logarithm of the structure parameters. The horizontal straight line represents a thickness deviation at one standard deviation. Thus, all the points below the horizontal line represent printed samples that have heights within one standard deviation of the ideal height, which is represented by the SiO_2_ prints. Likewise, all the points above the horizontal line represent printed samples that have heights less than one standard deviation of the ideal height. Thickness deviations above this line mean that the print failed and that the ink does not have the rheological properties to support the spacing ratio. Linear extrapolations were conducted for each formulation to relate the thickness deviation to the structure parameters. Finding each ink’s linear relationship led to the determination of the maximum spacing ratio $\eta_{max}$, which is the spacing ratio that produces a print with the thickness deviation equal to one standard deviation. Each formulation thus had a set of coordinates $\left(\eta_{max},{Κ}_{ink}\Psi_{lattice}\right)$ that were plotted, and a logarithmic curve was fitted to the data. The resulting empirical relationship is given as Equation (7), where the structure parameters must be in the region between that of SiO_2_ and the fitted model. Hence, this relationship demonstrates that based on the rheological properties of an ink, it can be determined how much spacing can be introduced during 3D printing for an FCT structure. Figure 5b presents ${Κ}_{ink}\Psi_{lattice}$ against the spacing ratio for the five formulations in which rheology was performed.

**Figure 6:**
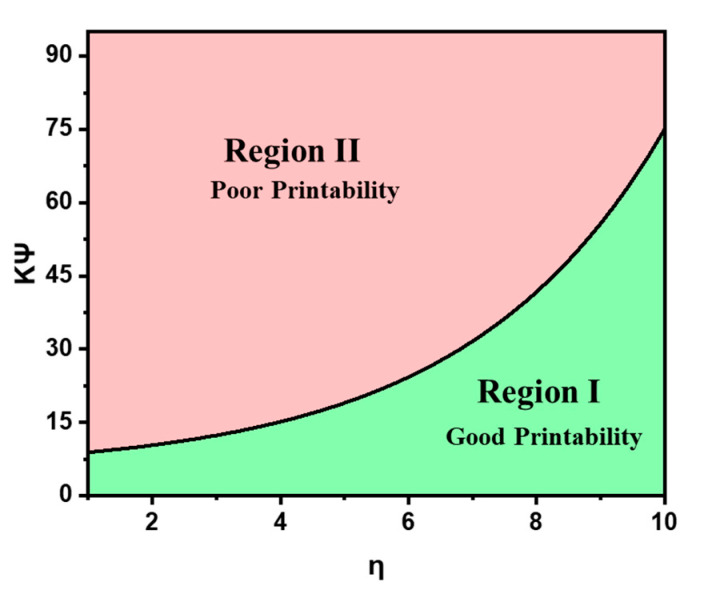
Using a 250 μm nozzle and an FCT geometry, rheology-tested ink formulations were assessed based on their material properties and thickness deviations to determine Equation (7), which provides a regime for printability. Values below the upper limit in Equation (7) are in Region I (Good Printability), which is defined as minimal slumping and within one standard deviation. Values above the upper limit are in Region II (Poor Printability), which is defined as thickness deviations above one standard deviation.

From these data, a printability profile was developed which shows regions of poor and good printability (Figure 6). Equation (7) defines the boundary of these regions, where good printability means that for a 250 μm nozzle printing an FCT part with a certain ink formulation, the resulting thickness will be within one standard deviation. A poor printability in this context means that the ink formulation will slump and have thickness deviations greater than one standard deviation. Using Equation (7), the ink parameter and maximum spacing ratio are provided for the rheological-tested ink formulations in Table 3.

(7) $${Κ}_{ink}\Psi_{lattice}\leq 2.7686e^{0.323\eta }+5.056$$

### 3.4. Thermomechanical Properties

**Figure 7:**
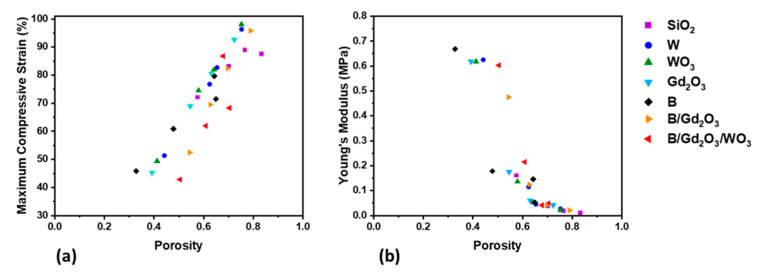
Mechanical properties from compression testing as functions of sample porosity: (**a**) maximum compressive strain, and (**b**) Young’s modulus.

Each of the printed cylinders underwent compressive strain testing, where the protocol utilized four compression and decompression cycles reaching a maximum load of 0.4 MPa. Taking the last cycle for data analysis, maximum compressive strains were determined and each sample’s Young’s modulus in the linear viscoelastic region was evaluated. Figure 7a,b show these material characteristics plotted against the porosities of the measured samples, respectively. From Figure 7a, it appears that the maximum compressive strains are bound within a region and increase in proportion to the porosity. This phenomenon is intuitive, where greater porosity in a viscoelastic material means that during compressions, more void spaces are filled in with material. This phenomenon is similar to what can explain Figure 7b, where Young’s modulus can describe how stiff or flexible a material is before it deforms. When all the samples are plotted together, the graph appears sigmoidal, where above a critical porosity, there is a dramatic decrease in the stiffness. This point seems to be approximately *φ_c_* = 0.55, where every formulation except for SiO_2_ had at least one sample below this amount. Notice that samples with porosities less than the critical porosity exhibited a Young modulus between 0.45 and 0.7 MPa, while those samples with porosities greater than the critical porosity exhibited a Young modulus between near 0 and 0.25 MPa. As the maximum load during the compression cycles was 0.4 MPa, this means that samples with porosities above the critical porosity were maximally compressed.

**Figure 8:**
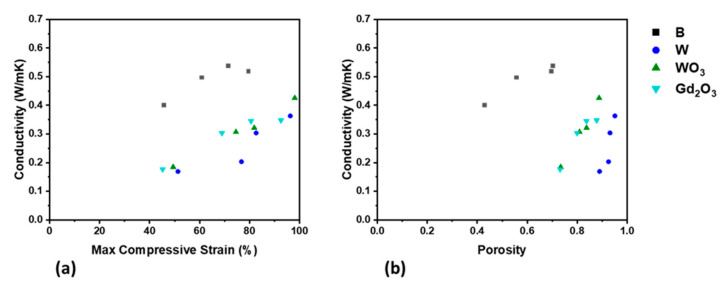
Thermal conductivity of 50 wt% metal- and ceramic-filled printed cylinders plotted against (**a**) porosity and (**b**) maximum compressive strain.

Thermal conductivity was explored in the formulations with 50 wt% metal or ceramic fillers, which include 3D-printed cylinders of B, W, WO_3_, and Gd_2_O_3_. Figure 8a,b present graphs of thermal conductivity against porosity and maximum compressive strain, respectively. The first observation from these figures is that thermal conductivity increases with porosity. As the maximum compressive strain and porosity are positively correlated, this means that thermal conductivity and maximum compressive strain should be positively correlated as well, which is demonstrated in Figure 8b. Additionally, the instrument that measures thermal conductivity applies a pressure of 60 psi ≈ 0.4 MPa and thus maximally compresses the samples with porosity greater than 55%. Therefore, greater contact is made between the printed layers, and heat can flow with fewer obstructions due to air voids. Another interesting point to take note of is how the thermal conductivities in Figure 8 are clustered together. Notice that B cylinders exhibit greater thermal conductivity than the others. This is due to the volume percent of the B ink; although all the formulations possess 50 wt% metal or ceramic filler, boron is less dense than the other fillers, and thus its volume fraction is much greater. This contributes towards a greater percolation of boron particles, where at 30 volume%, the heat flow has a less obstructed path due to the siloxane matrix and silica filler. On the other side, tungsten has a much greater density than tungsten (VI) oxide or gadolinium (III) oxide; thus, the volume percent of the filler in the W formulation (5.2%) is less than that of the WO_3_ (12.9%) and Gd_2_O_3_ (12.4%) formulations. This is overcome by the fact that metals, in general, have much greater thermal conductivities than ceramics, and this is indeed true when comparing tungsten (170 Wm^−1^K^−1^) to tungsten (VI) oxide (4.5 Wm^−1^K^−1^) or gadolinium (III) oxide (27 Wm^−1^K^−1^). Thus, despite the lower volume percent of the filler in W, the thermal conductivity is balanced out, and the W, WO_3_, and Gd_2_O_3_ printed cylinders exhibit similar thermal conductivity values.

One aspect of this work that must be pointed out is the blatant difference between many types of porosities. Throughout the literature, it is well documented that as porosity increases, many transport properties such as thermal and electrical conductivity decrease. Thus, a distinction must be made for micropores, which are voids within the material formulation itself, either by initial or process design, and structural voids, which are voids not in the ink but in the overall lattice structure. Although both types of pores would contribute towards increasing the compressive strain and thus increasing thermal conductivity, 3D printing offers a way to directly tune the porosity due to structural voids and therefore control the thermomechanical properties.

**Figure 9:**
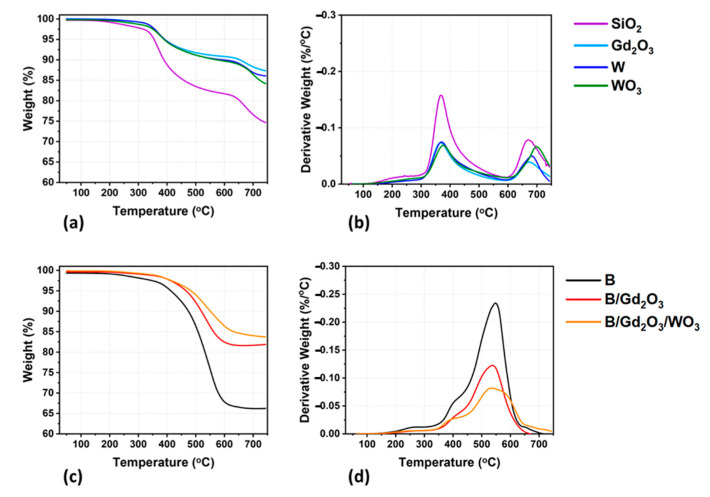
TGA curves for the 3D-printed cylinders: (**a**) weight percent for samples without boron; (**b**) derivative weight percent for samples without boron; (**c**) weight percent for samples with boron; and (**d**) derivative weight percent for samples with boron.

To understand the thermal limits of the printed parts, thermogravimetric analysis (TGA) was performed. Both the TGA and derivative TGA (DTGA) curves are shown for the printed parts in Figure 9. Data concerning the onset of thermal degradation, temperature of thermal decomposition, and residual mass for the printed parts are presented in Table 4. The formulations that do not incorporate boron are presented in Figure 9a,b, where two distinct degradation peaks occur. One interesting aspect is that the onset of thermal degradation *T_d5%_* of the SiO_2_ formulation occurs at a lower temperature than the others. Indeed, there is a 40 °C increase once the other fillers are added. Additionally, the residual mass of the parts with 4.5 wt% silica increases by 10 wt% when compared to the SiO_2_ formulation. Thus, the fillers increase the thermal stability of the ink. The first DTGA peak for all these printed formulations occurs around the same temperature, indicating the main pathway for thermal decomposition is unaltered. The TGA and DTGA curves for the samples containing boron are presented in Figure 9c,d. The B printed formulation demonstrates a 60 °C increase in *T_d5%_* compared to the SiO_2_ printed formulation, and this value increases further when multiple fillers are added. The printed formulation that has the highest *T_d5%_* is the B/Gd_2_O_3_/WO_3_ ink, which is 15 °C higher than the B/Gd_2_O_3_ ink and 130 °C higher than the SiO_2_ ink. Interestingly, the boron-containing printed parts exhibit only one main thermal decomposition peak, and it is shifted by an increase of 170 °C. As this is only observed in the ink formulations with boron, this indicates that boron affects the pathway of thermal decomposition. Indeed, a similar phenomenon has been observed before, where Rallini et al. reported that a polymeric matrix incorporating boron carbide resulted in a substantial shift in thermal stability towards higher temperatures [59]. It was proposed that this behavior resulted from the conversion to boron oxide and the subsequent inhibition of oxidation of the polymer matrix. In addition to the shift in thermal stability, a decrease in residual mass was observed when comparing the B and SiO_2_ formulations. The B printed parts exhibit a decrease in the residual mass, thus indicating that char products differ by way of B modifying the thermal decomposition chemistry. Overall, whenever fillers other than SiO_2_ were used, an increase in thermal stability was observed. Additionally, boron-containing inks, especially the ink that incorporates neutron and gamma shielding components, demonstrate the highest thermal stability of all the formulations.

### 3.5. Attenuation of Ionizing Radiation

**Figure 10:**
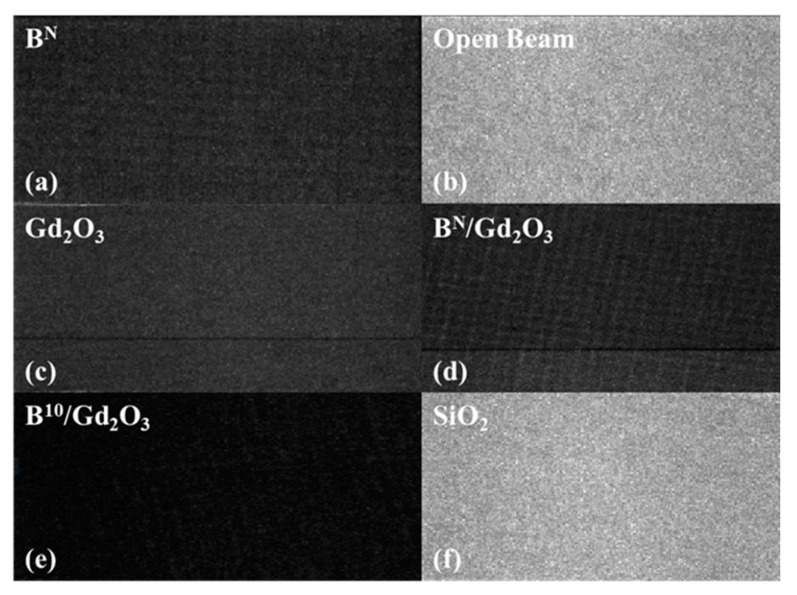
2D radiographs, summed over all neutron energies from 0.001 to 100 eV, of printed samples. For the formulations besides SiO_2_, the total filler content was 50 wt%. Lighter images correspond to more neutrons passing into the detector, while darker images correspond to a greater attenuation of incoming neutrons: (**a**) 50 wt% B^N^; (**b**) open beam (no sample); (**c**) 50 wt% Gd_2_O_3_; (**d**) 40/10 wt% B^N^/Gd_2_O_3_; (**e**) 40/10 wt% B^10^/Gd_2_O_3_; and (**f**) 10 wt% SiO_2_. All neutron-exposed pads were 3D printed using 500 um spacing.

Neutron radiography was performed on some printed samples to obtain a qualitative assessment of their ability to behave as radiation shields. Five ink formulations were used to print cylinders for this experiment: B^N^; Gd_2_O_3_; B^N^/Gd_2_O_3_; B^10^/Gd_2_O_3_; and SiO_2_. B^N^ in the formulations refers to natural abundance boron, which contains approximately 20% B^10^ and 80% B^11^. B^10^ in the formulations refers to isotopically enriched boron, which is nearly 100% B^10^. Besides the SiO_2_, all the formulations had 50 wt% non-silica filler. The B^N^/Gd_2_O_3_ and B^10^/Gd_2_O_3_ formulations contained 40 wt% boron and 10 wt% gadolinium (III) oxide. The qualitative results of the 2D radiography experiments are shown in Figure 10. The amount of light corresponds to the amount of neutrons that passed through the detector, which was being blocked by the printed cylinders. Thus, a lighter image corresponds to more neutrons passing through, while a darker image corresponds to less neutrons passing through. Therefore, the darker the image, the greater the printed cylinder behaved as a radiation shield. As it can be observed in the figure, the SiO_2_ cylinder did not attenuate much at all, while the other printed samples attenuated a significant amount of incoming neutrons. As expected, due to B^10^ being the isotope that absorbs neutrons, the B^10^/Gd_2_O_3_ sample provided the greatest shielding.

**Figure 11:**
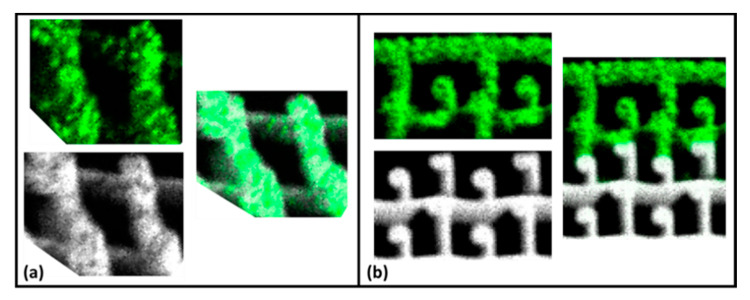
3D printing with formulations of WO_3_ and Gd_2_O_3_ (**a**) in one ink to create a homogeneous part and (**b**) as separate inks to produce a heterogeneous part.

After the neutron radiography experiments proved that the ink formulations performed successfully as shields, more carefully designed printed structures were developed to incorporate additional functionality. This was conducted by 3D printing multi-material heterogeneous cylinders using Gd_2_O_3_ and WO_3_ inks. As a comparison, homogeneous parts were also fabricated. For the homogeneous parts, Gd_2_O_3_ and WO_3_ were incorporated into the same ink formulation as already described and printed. Using X-ray florescence (XRF), a color map was generated, shown as Figure 11a, where the green coloration represents WO_3_, and the white coloration represents Gd_2_O_3_. Observe that both colorations are superimposed, and thus the fillers exist in a homogeneous distribution within the printed part. This is in contrast to Figure 11b, where separate formulations of Gd_2_O_3_ and WO_3_ were used to print a single part where they did not exist in the same space, thus exhibiting a heterogeneous distribution. Although multi-material 3D-printed structures have been explored [19], this further demonstrates that a single printed part for radiation shielding can be constructed from a variety of ink formulations to possess multifunctional characteristics.

## 4. Conclusions

Radiation shielding DIW 3D printing formulations were developed, optimized, characterized, and used for 3D printing. Varying spacing ratios, which are the ratios of the center-to-center distance between adjacent struts to the diameter of the printed struts, were used when printing face-centered tetragonal (FCT) lattice structures. A height deviation was observed in the final printed cylinders when compared to the theoretical design, which was a result of the ink rheological properties. Previous models that correlate rheology and printability were found to not accurately predict the observations in this study, and as such, a new model was needed. Using beam deflection as a starting point for a model, an empirical relationship was created that correlated the maximum printable spacing ratio with the structure parameter, which is defined as the product of the ink and lattice parameters. Thus, using this empirical model allows for the refinement of new ink formulations in the future by way of relating the rheological properties of an ink to the quality of its final printed structure.

By varying the spacing ratio of the printed cylinders, the thermomechanical properties of printed parts were able to be altered and thus characterized. Increasing the spacing ratio led to an increase in porosity, which results in an increase in the maximum compressive strain, a decrease in Young’s modulus, and an increase in thermal conductivity while compressed. Furthermore, the thermal stability of the samples was assessed, and it was found that incorporating fillers in addition to silica increased the thermal stability of the printed cylinders. When boron was used as a filler, the pathway of thermal decomposition was altered as well, leading to formulations that possessed much greater thermal stability than the other printed formulations.

Neutron radiography proved that the parts behaved successfully as radiation shields, and thus further developments and refinements can be made to the ink formulations for specific applications. Furthermore, heterogeneity in printing these radiation shields was tested, where an ink formulation combining WO_3_ and Gd_2_O_3_ was used to print a homogeneous cylinder, and two different ink formulations, one containing WO_3_ and the other containing Gd_2_O_3_, were used to print a heterogeneous cylinder. Using X-ray fluorescence (XRF), a color map of the elements was generated and demonstrated that the printed cylinders were indeed homogeneous and heterogeneous. Thus, this works provides a foundation for further research into developing greater tunable DIW inks that can be used for multi-ink-specific heterogeneous 3D printing for a variety of specialty applications.

## Figures and Tables

**Table 1:** The formulations developed and their densities along with the weight and volume percent of each filler component.

		Weight Percent (*w*/*w*)/Volume Percent (*v*/*v*)					
		SiO_2_	W	WO_3_	Gd_2_O_3_	B	Density (g/cm^3^)
**Formulation**	**SiO_2_**	10/4.3	0/0	0/0	0/0	0/0	1.064
	**W**	4.5/3.6	50/5.2	0/0	0/0	0/0	2.004
	**WO_3_**	4.5/3.3	0/0	50/12.9	0/0	0/0	1.842
	**Gd_2_O_3_**	4.5/3.3	0/0	0/0	50/12.4	0/0	1.850
	**B**	1.5/0.9	0/0	0/0	0/0	50/30	1.424
	**B/Gd_2_O_3_**	1.5/1.2	0/0	0/0	30/8.1	40/33.7	1.998
	**B/Gd_2_O_3_/WO_3_**	1.5/1.2	0/0	20/5.6	10/2.7	40/33.7	1.995

**Table 2:** Rheological properties of a representative sample of ink formulations.

Formulation	$\mathrm{Equilibrium}\;\mathrm{Storage}\;\mathrm{Modulus}\;({G^{\prime }}_{eq})\;(\mathrm{Pa})$	$\mathrm{Yield}\;\mathrm{Stress}\;(\sigma_{y})\;(\mathrm{Pa})$	Flow Point (Pa)
**SiO_2_**	55,470	3650	4610
**W**	42,600	3390	3900
**WO_3_**	57,400	2940	4480
**B**	78,720	400	2270
**B/Gd_2_O_3_**	2,277,190	5330	11,050

**Table 3:** Ink parameters for the rheological-tested ink formulations and their maximum spacing ratios according to Equation (7).

Formulation	Κ*_ink_* (m^−1^)	*η_max_* (From Equation (7))
**SiO_2_**	4.345 × 10	7.7
**W**	7.286 × 10	6.0
**WO_3_**	1.200 × 10^2^	5.0
**B**	6.874 × 10^3^	1.5
**B/Gd_2_O_3_**	1.571 × 10^3^	2.2

**Table 4:** Thermal stability properties of the 3D-printed formulations.

Formulation	*T_d5%_* (°C)	*T_dMax_* (°C)		*m_f_* (%)
**SiO_2_**	355	371	675	75
**W**	392	373	680	86
**WO_3_**	390	377	701	84
**Gd_2_O_3_**	392	369	675	87
**B**	413		548	66
**B/Gd_2_O_3_**	467		536	82
**B/Gd_2_O_3_/WO_3_**	483		537	84

## Data Availability

The authors confirm that the data supporting the findings of this study are available within the article.

## Supplementary Materials

The following are available online at https://www.mdpi.com/article/10.3390/polym13193284/s1, Figure S1: Storage and loss moduli of the rheology-tested ink formulations during the stress sweeps.
