# Supplementary material for: Tuning the 3D Printability and Thermomechanical Properties of Radiation Shields
Source: Polymers (Basel). 2021 Sep 26;13(19):3284. doi: 10.3390/polym13193284 (PMC8512519; doi:10.3390/polym13193284)
Supplement: Supplementary file 1 [file polymers-13-03284-s001.zip › polymers-1392824-supplementary.pdf]

Supplemental information

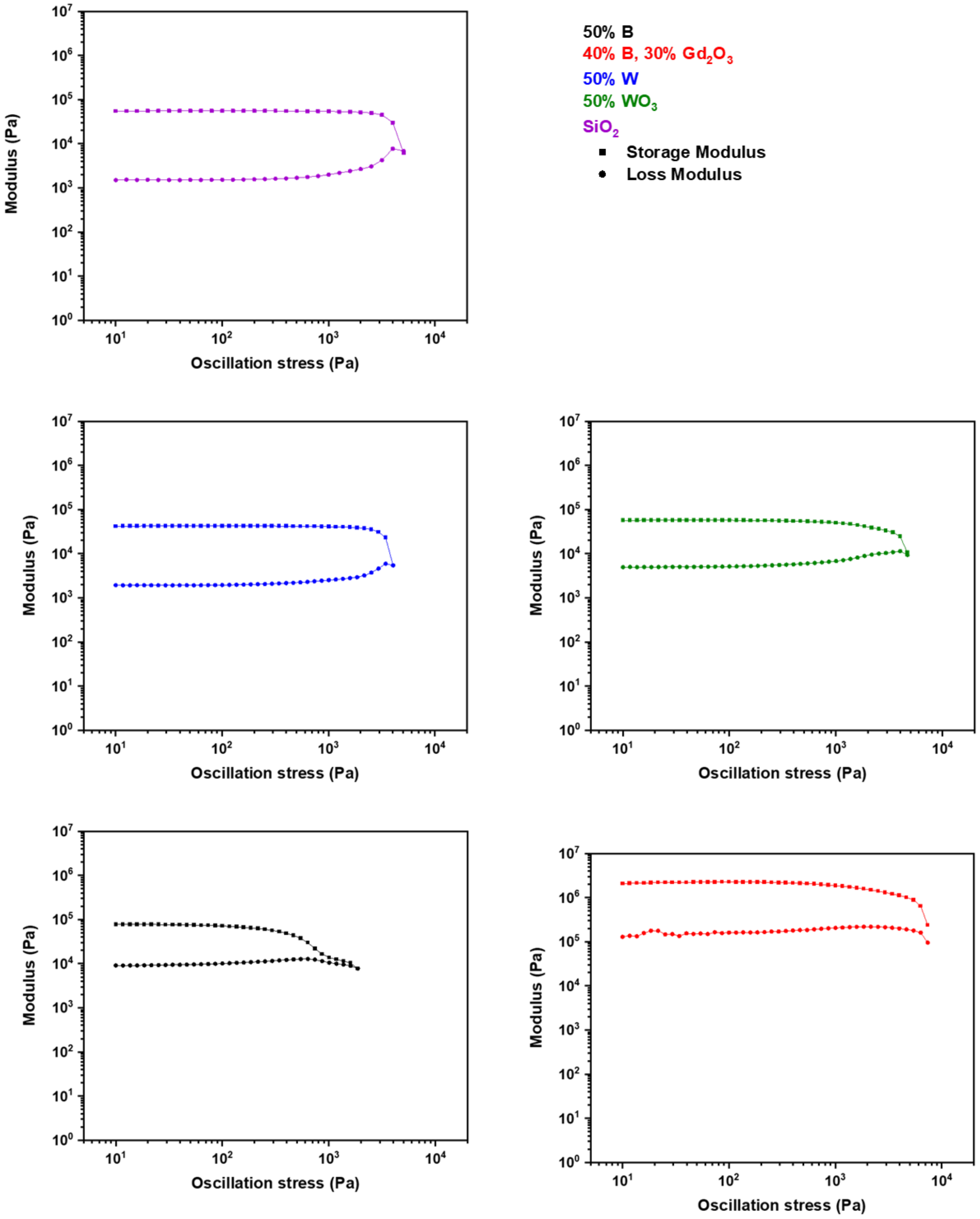

Figure S1: Storage and loss moduli of the rheology-tested ink formulations during the stress sweeps.

FCT geometry theoretical porosity calculation:

Consider a rectangular prism constructed of layers of 3D printed struts. The struts have a diameter *d* and the center-to-center distance between the struts is *L*. The first layer comprises *N* printed struts oriented in the same direction and each layer is oriented orthogonal to the layers printed directly above and below. Furthermore, every other layer is staggered, where the printed struts are in-between those from two layers above and below. The figures below demonstrate this construction.

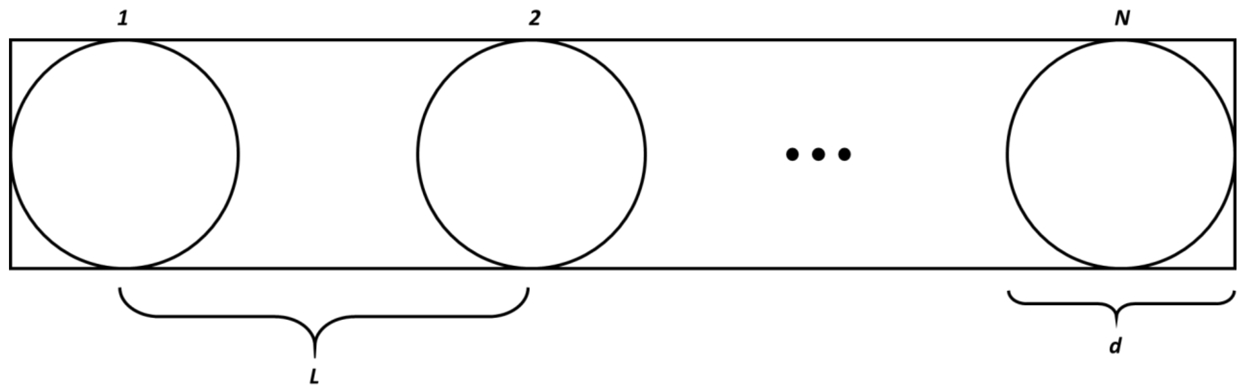

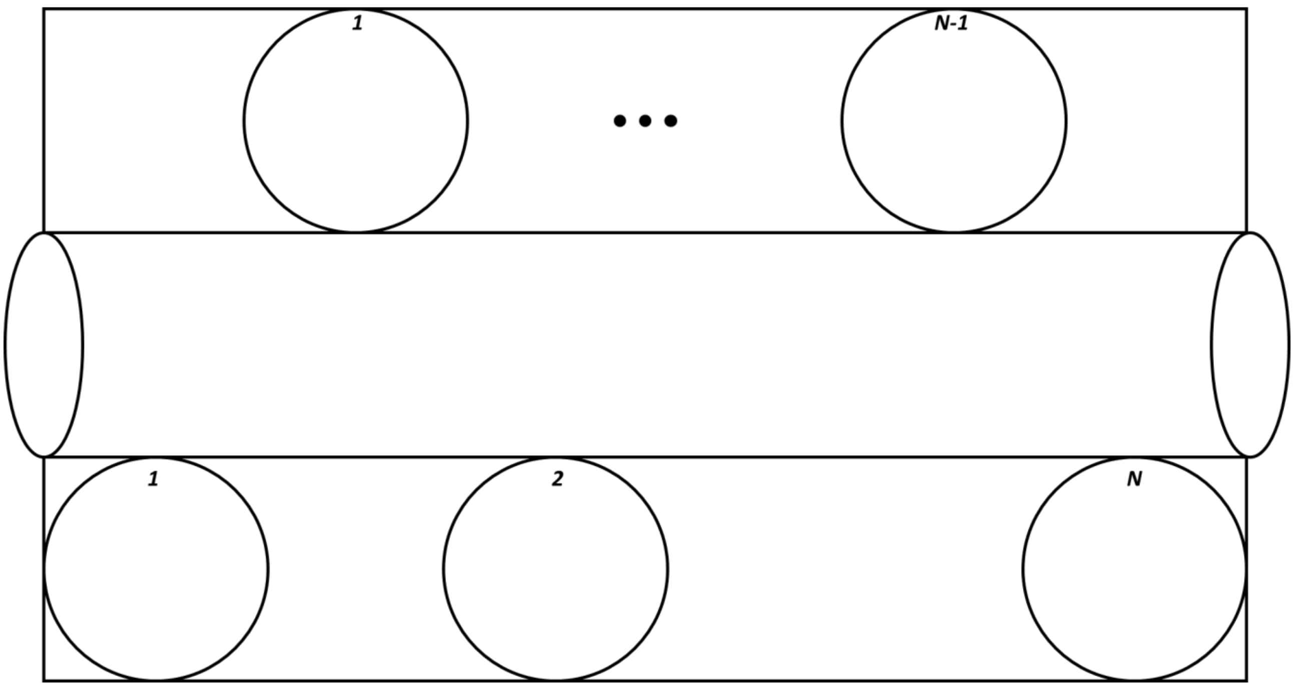

Thus, the height, width, and length of each layer is *d*, *(n-1)L+d*, and *(n-1)L+d*, respectively. Due to the staggered nature of the lattice, every four layers repeats. Therefore, only considering

four layers, where the first two have *N* struts and the last two have *N-1* struts, the porosity can be evaluated by comparing the volumes of the struts against the total control volume. The volume of a printed strut is that of a cylinder $V_{strut} = \frac{\pi}{4} d^2 \big((N-1)L + d\big)$, whereas the volume of a layer is that of a rectangular prism $V_{layer} = d\big((n-1)L + d\big)^2$.

Because the porosity is the empty space, $\varphi_{FCT} = 1 - \frac{\big(2n+2(n-1)\big)V_{strut}}{4V_{layer}} = 1 - \frac{(2n-1)\pi d}{8\big((n-1)L+d\big)}$, the idealized porosity of the FCT geometry can be assessed by taking the limit as the number of struts in a layer goes to infinity $\varphi_{FCT}^{\infty} = \lim_{n\to\infty} \varphi_{FCT} = 1 - \frac{\pi d}{4L} = 1 - \frac{\pi}{4\eta}$, where $\eta = {}^{L}/_{d}$ is the spacing ratio for the lattice structure.
